# An Autonomous Wearable System for Predicting and Detecting Localised Muscle Fatigue

**DOI:** 10.3390/s110201542

**Published:** 2011-01-27

**Authors:** Mohamed R. Al-Mulla, Francisco Sepulveda, Martin Colley

**Affiliations:** School of Computer Science and Electronic Engineering, University of Essex, Colchester, UK; E-Mails: fsepulv@essex.ac.uk (F.S.); martin@essex.ac.uk (M.C.)

**Keywords:** muscle fatigue, sEMG, feature extraction, classification

## Abstract

Muscle fatigue is an established area of research and various types of muscle fatigue have been clinically investigated in order to fully understand the condition. This paper demonstrates a non-invasive technique used to automate the fatigue detection and prediction process. The system utilises the clinical aspects such as kinematics and surface electromyography (sEMG) of an athlete during isometric contractions. Various signal analysis methods are used illustrating their applicability in real-time settings. This demonstrated system can be used in sports scenarios to promote muscle growth/performance or prevent injury. To date, research on localised muscle fatigue focuses on the clinical side and lacks the implementation for detecting/predicting localised muscle fatigue using an autonomous system. Results show that automating the process of localised muscle fatigue detection/prediction is promising. The autonomous fatigue system was tested on five individuals showing 90.37% accuracy on average of correct classification and an error of 4.35% in predicting the time to when fatigue will onset.

## 1. Introduction

Muscle fatigue is a reduction of the ability of a muscle to contract and exert force. Localised muscle fatigue can be both beneficial in promoting muscle growth as seen in body-builders or harmful causing serious injury when the level of fatigue is high. When muscle fatigue is not detected soon enough, it can often inflict injuries [1], causing not only pain to the subject but often a financial burden as well [2]. The psychological aspects also affect the physical fatigue phenomenon, as people may feel physiologically weaker than they really are, which in turn can affect their physiological performance.

Research has mainly focused on localised muscle fatigue as the process of a decline in the force during a sustained activity [3], which gives a definition of fatigue as the inability to exert any more force or power. Barry argues that this definition indicates that fatigue occurs rapidly after the onset of a sustained exercise although the subject may be able to sustain the activity. However, the impairment in the muscle will eventually lead to total fatigue, where it is impossible to continue the task [4].

An autonomous device is also referred to as a smart device, which means it is digital and computer networked. By being autonomous, the device is self-governed and will work without human interference. Numerous wearable devices have been developed for different purposes, although the majority are designed for use within the healthcare field. Researchers have used different kinds of sensors in their wearable devices, however, most of them have made use of sEMG electrodes, either on their own or in combination with other sensors. sEMG electrodes are fairly inexpensive and can be easily placed on various muscles in the body, making them suitable for a variety of purposes.

Wearable computing has been applied in various fields, in particular within the healthcare sector to monitor the health and the welfare of patients, where movements and behaviours are studied. It has been implemented in the use of prosthetic control devices where the movements of the prosthetic device are controlled by sEMG signals from muscles elsewhere in the body. Naik *et al.* identified hand gestures and unvoiced speech commands using sEMG signals recorded from different muscles as a means for human-computer interaction [5]. In a study by Bu *et al.*, two sEMG sensors were placed in a mask to measure facial movements, and it was found that the signal from a piezoelectric thin film sensor is capable of controlling human interfaces [6]. Constanza *et al.* demonstrated that motionless gestures can be used to control realistic, multimodal interfaces [7]. In such systems, users wear an intimate armband with built-in sEMG electrodes, which senses isometric muscular activity. Amft *et al.* have developed an automated dietary monitoring (ADM) system which recognises food intake and eating behaviours [8]. This system made use of the sound of swallowing and EMG signal modalities for sensor pattern recognition. In similar systems on-body sensors and ambient sensor networks have been combined to monitor food intake, where radio-frequency identification tags are used to label food and worn on subjects’ hands [9,10], in addition to video monitoring and weight-sensitive tables [10,11]. Another form of wearable devices is body area network (BAN) mainly used for health systems in monitoring patients, where various communication devices are worn on the body to provide personalised services for the user. Such systems make use of sEMG sensors, a local biofeedback device and a personal data assistant, which receives and processes data from the sensors and uses WiFi, GPRS and/or UMTS to transmit the data to a remote health professional [12,13].

Few pieces of research have been conducted to this day on using autonomous devices for detecting/predicting muscle fatigue. To evaluate patients with lower back pain, Chhikara *et al.* developed a multi-sensor wearable device for monitoring disability in movement of the lumbar spine and pelvis, and sleep disturbances due to the disability, in addition to muscle fatigue and activity pattern [14]. Their system uses a combination of inertial sensors placed on the lower back and pelvis, a light sensor located on the chest and sEMG sensors placed on back muscles and buttock muscles, which in a future study will all be placed in a lumbar belt or in wearable textiles to facilitate usability. A ubiquitous wearable unit for controlling muscular fatigue during cycling exercise sessions was developed by Kiryu *et al.* and proved sufficient in controlling the workload in an exercise machine to prevent the occurrence of fatigue [15,16].

Classification of localised muscle fatigue can be carried out using various classification methods, although they are not highlighted in the literature on muscle fatigue research. OCAT is a classification function developed by Torvik *et al.* in 1999, where the aim was to create a flexible, but simple prediction function [17]. In their study on predicting if a muscle is fatigued or rested by investigating the peaks and characteristics fractile frequencies in the EMG signals, they found, in their comparison with other classification methods, that OCAT achieved the highest accuracy. In the testing set, the classification accuracy of OCAT was 89.1%, compared with 84.8% for logistic regression, while fuzzy k-nearest classification obtained an accuracy of 82.6%. ANN also gave promising classification accuracy, although it fluctuated from 80.4–89.1%.

The detection and classification of muscle fatigue adds important information to the fields of human-computer interactions, sport injuries and performance, ergonomics and prosthetics. An automated system that will predict and detect when fatigue occurs is especially useful in sports related scenarios, where fatigue can lead to injury. The system will guide the user in his training and act as a warning device, to avoid unnecessary strain on the muscle, promoting improvement and preventing injury. This system can also be applied in occupational health and ergonomics, in particular where there is a risk of work-related musculoskeletal disorders. Localised muscle fatigue in the work place may cause injury, in particular if a task causes elevated static muscle activity [18]. An automated system can predict when the muscle is fatiguing and hence avoid injury in this scenario. Similarly, in ergonomics, a system like this can aid in the correction of posture problems before the occurrence of muscle strain or injuries.

Muscle fatigue is a physiological phenomenon which can only be precisely measured by invasive means. This is unsuitable for daily applications. However, surface electromyography (sEMG) has been known to carry fatigue related information for at least 40 years [19,20], making it a suitable, if not as precise, means for detecting muscle fatigue non-invasively. Therefore this study has focused on signal acquisition of localised muscle fatigue using sEMG, while the kinematic aspects are measured using a goniometer. The goniometer provided the bases in training the sEMG classifier as it proved to be a reliable indicator of fatigue. Almulla *et al.* carried out a study to enable predicting the time to fatigue by using an evolved feature that combined many known sEMG fatigue features using a GA [21]. Although that study was performed with pre-recorded signals and lacked the autonomous real-time factor, which has been developed in this study.

The aims of this study is to automate the detection and prediction of fatigue by detecting the onset of Transition-to-Fatigue and its progression using the 1D spectro feature [22] followed by predicting the time to fatigue which is based on the onset of Transition-to-Fatigue detection [21]. The autonomous system will also provide visual feedback to the end user informing them on the fatigue status of the muscle.

In order to achieve these aims a test bed for the automation was developed. The SunSPOT (Sun Small Programmable Object Technology) by Sun Microsystems was used with additional amplification and filtering circuitry to enable the real-time acquisition/processing of the signals.

## 2. Methods

The first stage of this research was to create the autonomous system, and the second stage was to conduct experiments to train and test the developed autonomous system by acquiring the sEMG emanating from the biceps brachii muscle and the kinematic aspects of every subject. This stage involved extracting the 1D spectro feature from the sEMG followed by labelling the extracted feature into two classes using the kinematic aspects of muscle fatigue, such as elbow angle and arm oscillation into a fuzzy classifier, obtained by a goniometer. The process of labelling was solely used for training the classifier and to measure the classification accuracy of the extracted sEMG feature during the testing phase. Finally, training of the classifier was undertaken followed by testing its classification performance.

### 2.1. Experimental Set-Up

The data were collected from five male athletic, healthy subjects (mean age 28 +/− 2.5 year), all non-smokers. The five participants were willing to reach physical fatigue state but not a psychological one. Physical fatigue manifests itself by changes in physiological processes, while psychological fatigue is influenced by subjective factors, e.g., lack of motivation and/or tiredness [23]. Chaffin [24] used the term localised Muscle Fatigue (LMF), as one example of physiological fatigue, which refers to the inability of a given muscle to maintain a desired force, which is the definition of fatigue used in this study. The participants were seated on a preacher biceps curl machine to ensure stability and biceps isolation while performing a static biceps curl activity. Once the participants reached total biceps fatigue (not able to exert force) they stopped. As the subjects were athletes they are familiar with fatigue, however, they were encouraged by the experiment team to continue exerting force for as long as possible. For each of the five participants, three trials in total where carried out (two trials for training and one for testing). In all the trials we used 30% Maximum Voluntary Contraction (MVC) for each subject. There was a resting period of five days for each of the three trials ensuring full recovery of the biceps brachii. The study was approved by the University of Essex’s Ethical Committee and all subjects signed an informed consent form prior to taking part in the study.

In this study two types of sensors where used for signal acquisition; goniometer and sEMG electrodes. The goniometer measured the angle between the elbow joints, where the subjects should maintain an angle of 90°. A flexible electro-goniometer (Biometrics Ltd.) was placed on the lateral surface of the dominant arm’s elbow to measure the elbow angle, with adhesion on the areas distal and proximal to the joint. An important consideration in selecting the most appropriate goniometer sensor is that it must be capable of reaching across the joint, so that the two end blocks can be mounted where least movement occurs between the skin and underlying skeletal structure. The goniometer has been used in combination with EMG, mechanomyography (MMG) and ultrasound in various muscle groups to measure localised muscle fatigue [25–31].

sEMG electrodes (Comepa 30mm disposable electrode) were placed on the participants biceps brachii lower belly avoiding the estimated innervation zone and toward the distal tendon to acquire sEMG reading. The myoelectric signal was recorded using two channels, Single Differential (SD) electrodes, with the (Sun microsystems sunspot) A/D conversion at 1,000 samples/s. The sEMG signals acquired during the experiment were only filtered with a dual pass Butter-worth filter, with the fifth order passband being between 1 and 500 Hz. The goniometer readings were also acquired simultaneously. The goniometer provided a reliable mechanical indication on the development of fatigue. Classical biceps muscle fatigue for the clinically healthy individuals usually manifest itself by small oscillation or vibrations followed by a difficulty in maintain a task [32,33], in our case the 90° elbow angle. A reference electrode will ensure a common reference to the differential input of the pre-amplifier in the electrode, which was was placed on the non-dominant arm on a bony part where there is no muscle, in our case the hand wrist.

### 2.2. Hardware Overview

**Figure 1.**
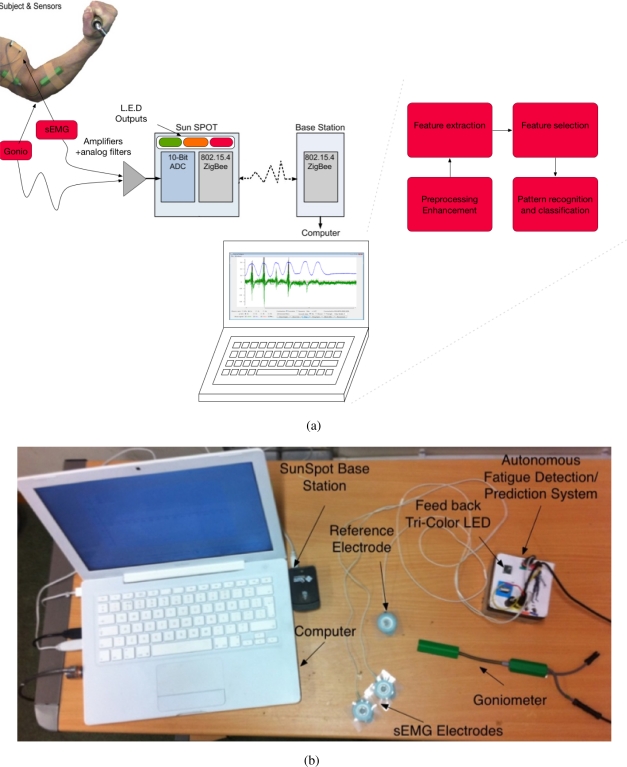
An overview of the hardware architecture and the actual system components. **(a)** The Autonomous fatigue detection and prediction system setup; **(b)** System Components.

**Figure 2.**
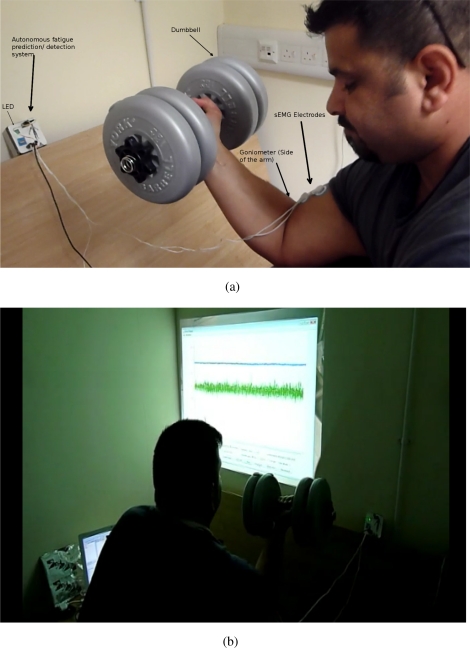
Subject During a trial. **(a)** Subject during a testing trial, showing the subject with the autonomous device; **(b)** During a testing trial.

The autonomous fatigue detection/prediction system consists of two main parts: the amplifier with the filtering circuit and the SunSPOT. The amplifier amplifies the signals emanating from the biceps brachii. The SunSPOT uses the Analogue to Digital Converter (ADC) on-board to sample the analogue signals (sEMG and the goniometer), converting them to digital signals, thus enabling the processing. The SunSPOT also provides an on-board set of LEDs which are used in giving feedback to the subject on the status of the tested muscle. Figure 1(a) and 1(b) shows an overview of the hardware architecture and the actual system components. Figure 2(a) and 2(b) shows a snapshot of one of the testing trial that was conducted.

### 2.3. The Amplifier and Filters

The system uses a differential pair amplifier with inputs from two leads coming from the muscle, in this case the biceps brachii. The signal’s amplitude range is from 0 to 10 mV (peak-to-peak), which means amplification is need to ensure the 10-bit ADC at 3.3 V can properly convert the voltage to a usable signal. The amplification of the sEMG signal was about 330 times and the goniometer was amplified by 270 times. The hardware filtering of the sEMG signal was bandpassed with 10 to 1,000 Hz.

### 2.4. SunSPOT

An embedded platform called SunSPOT is produced by Sun Microsystems, which includes an Atmel Atmega88 microcontroller and a 10-bit ADC. The board has two analogue input pins that will sample into the ADC. The ADC, which is 10-bit, uses the SunSPOTS internal 3.3 V battery as the power source. Thus, the SunSPOT has steps of 3.22 mv and the signal is amplified so that it is clearly readable. The sampling rate of fatigue related contents of the sEMG signal is 1,000 Hz. The SunSPOT is capable of sampling up to 22 MHz, due to Java Technology, however, it can sample at around 1.5 KHz which is still more than necessary. The SunSPOT also provides eight Tri-color LEDs which are used in this system to provide the subjects’ status of the muscle being inspected. The colours on the device will change to green for Non-Fatigue, orange for Transition-to-Fatigue and red for Fatigue, according to the trained classifier.

### 2.5. Surface Electromyography (SEMG) and Kinematics

Current research tends to focus on two classes of localised muscle fatigue, Non-Fatigue and Fatigue. Fatigue relates to the onset of fatigue during a muscle contraction, while Non-Fatigue is the state of the muscle during the contraction that occurs before the onset of fatigue. However, our research group has suggested an additional, third class of fatigue, Transition-to-Fatigue [32], highlighting that attempting to predict the onset of fatigue class at the time at which it occurs is insufficient since then the predictive nature of the measurement is inherently lost. Rather, once the onset of Transition-to-Fatigue is detected, what follows is a progressive process until fatigue onset. In the first stage of fatigue, Non-Fatigue, the fresh muscle is able to exert its maximum force. Once the fresh muscle starts to fatigue, new recruitment of muscle fibres occurs, and this is usually manifested as ON, where there is a sudden increase in motor unit action potential (MAUP) firing rate (onset of Transition-to Fatigue). After this increase, a progression of this state (Transition-to-Fatigue) is observed until the onset of Fatigue, where less myoelectric power can be detected emanating from the muscle due to loss in conduction velocity (CV) within the localised muscle.

**Figure 3.**
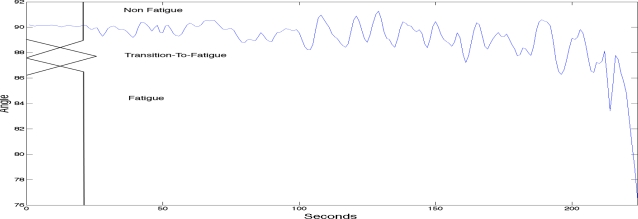
The use of elbow angle to label and classify the signal.

**Figure 4.**
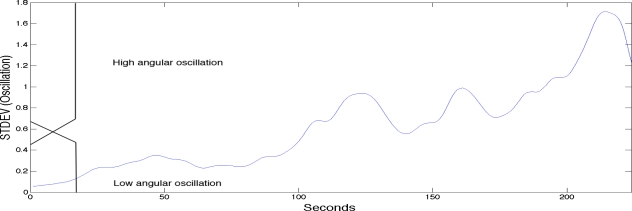
The use of angular oscillation to label and classify the signal.

To label the sEMG signals into the three classes of fatigue as explained above a fuzzy classifier was used. The fuzzy classifier was setting the boundaries for labelling the sEMG signals with the three classes of localised muscle fatigue. The fuzzy classifier automated the classification process and was based on two main kinematic criteria; elbow angle and arm oscillation. As with most fuzzy classifiers a single output is generated providing the classification. See Table 1 for the rule base. Figure 3 shows the first fuzzy set input which is the elbow angle provided by the goniometer (0 to 180 degrees). The figure also has a superimposed illustration of a single goniometer trial signal giving an example of how the fuzzy classifier is finding the boundaries to enable the labelling of the sEMG signal. Figure 4 shows the second fuzzy set input which is the arm oscillations (*i.e.*, the standard deviation of the elbow angle), also provided by the goniometer. An increase in the standard deviation of the goniometer signals indicates high angular oscillation. The increase in oscillations is indeed a precursor of physiological fatigue [22,32,34].

## 3. sEMG Signal Analysis and Feature Characterisation

**Figure 5.**
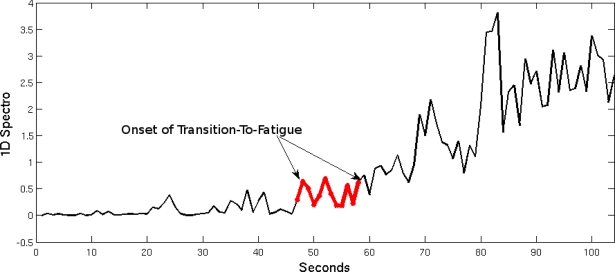
The 1-D Spectro feature, illustrating the onset of Transition To-Fatigue by the fuzzy classifier.

A one-dimensional spectrogram (1D Spectro), which is a composite feature, was developed in a previous study to assist in the prediction and detection of muscle fatigue, in particular the onset of Transition-to-Fatigue [22,34]. It is thought that when both the instantaneous median frequency (IMF) and the total band power are unified by subtraction they produce a feature that imitates a spectrogram (containing time and frequency contents) and simplifying it to its one-dimensional (1D) form. The 1D spectro is light on the resources, however it is still reliable in real-time prediction and detection of fatigue. It was established in previous research that once the onset of Transition-to Fatigue has taken place the output of the 1D spectro will increase [22]. This directly correlates to the fuzzy classifier that was used to label the signal. Figure 5 contains the Non-Fatigue and Transition-to-Fatigue segments. The boundary where the onset of Transition-to-Fatigue is understood is marked in the figure.

As mentioned above, the 1D spectro is created by unifying the signal by subtracting the total band power and the IMF. These features are explained below.

The total band power of the sEMG signal can be estimated using Welch’s method. This method has been used in several sEMG fatigue analysis studies [35] and has proved to be useful in quantifying the power of the EMG signals. The spectral frequency can be redefined to represent the non-stationary nature of the signal, or the instantaneous frequency of the frequency content of the signal [36]. The instantaneous median frequency (IMF) was introduced by Roy *et al.*:

(1) $$\int_{0}^{\mathrm{IMDF}(t)}P(t,w)\mathrm{dw}=\int_{\mathrm{IMDF}(t)}^{\infty }P(t,w)\mathrm{dw}$$

where *t* is time, *P* is the PSD function, *w* is the radian frequency and *d* is the differential operator [35].

Studies by Oskoei *et al.* concluded that a significant decline in the IMF of the signal is a significant manifestation of fatigue occurrence [37]. In addition, Georgakis *et al.* demonstrated that the average instantaneous frequency is superior to the mean and median frequencies for the analysis of muscle fatigue during sustained contractions [38].

## 4. Classification

In this study we are mainly interested in a simple and fast algorithm to be used by the autonomous system in classifying the two states of fatigue (Non-fatigue and Transition-to-Fatigue), and it was found that the Linear Discriminant Analysis (LDA) is most appropriate for this case while retaining exceptional performance. To train and test the classification performance we used three trials in total for each subject. Two trials where used for training and one trial was used for testing. In training the classifier, the sEMG 1D spectro feature was extracted from the sEMG, which was then labelled with the fuzzy classifier described in Section 2.5 with the appropriate class (Non-fatigue or Transition-To-Fatigue). Once the training of the classifier is complete and manually checked to ensure correct training, the testing phase was carried out. In the testing phase the sEMG was classified based on the training while the fuzzy class labeller was only used to measure the performance of the classification providing the percent correct classification over time (see Figure 7 in result section). The following linear transformation describes the classification where the LDA maps the data (feature vector) x:

(2) $$y=w^{t}x+w_{0},$$

where w and w0 are determined by maximising the ratio of between-class variance to within-class variance to guarantee maximal separability.

## 5. Time to Fatigue Prediction

**Figure 6.**
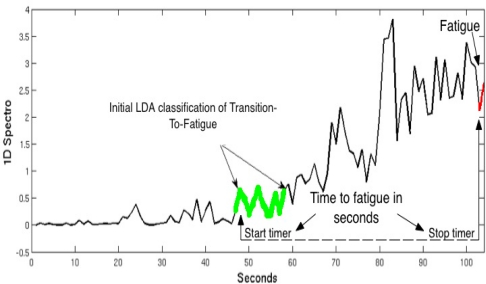
Illustration indicating were in time the onset of Tranition-To-Fatigue and the onset of Fatigue occurred during a trial.

In order to predict the time to fatigue we have used a simplified version of the algorithm used by Almulla *et al.* [21], as the current study is only focusing on a single MVC. In a previous study, Almulla *et al.* trained the ANN (Artificial Neural Network) based on the positive correlation between percentage of MDS (Maximum Dynamic Strength) (40% or 70%) and the time in seconds in which the subject fatigues. In this paper we have simply used the LDA classifier as a marker to start a timer measuring the number of seconds to when the subject fatigues from the initial detection of the onset of Transition-to-Fatigue. Determining the onset of the Transition-to-Fatigue class is essential; once the onset of this class is detected by the LDA classifier it was found on both training sets, for all five subjects, that the time (number of seconds) between the onset of Transition-to-Fatigue and Fatigue differ slightly (see Table 3) in the results section. Figure 6 shows an illustration of one of the trials pointing the time where the LDA started classifying the signal as Transition-to-Fatigue. As soon as the Transition-to-Fatigue is detected by the LDA a timer starts as shown in the diagram after which the time when the subject has fatigued is also recorded.

## 6. Results

Table 2 shows the correct classification in percent of the LDA classifier when classifying Fatigue and Transition-to-Fatigue. It can be seen that the mean correct classification is 90.37% while the highest correct classification is 93.09. In addition, the table shows a relatively low standard deviation of 3.22, which indicates stability of the system giving repeatable results across our subjects.

**Figure 7.**
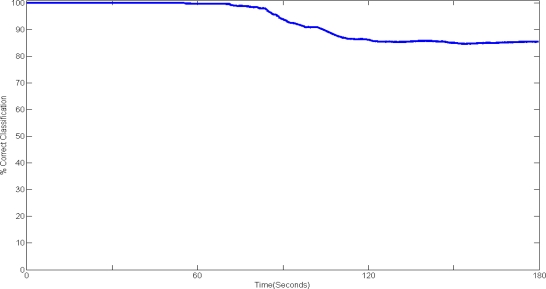
Classification over time during one of the testing trial.

Figure 7 above shows the classification performance over time during a single test of the system.

It can be seen that classification up to fifty five seconds shows high classification accuracy indicating that the algorithm is classifying the Non-fatigue portion of the sEMG optimally, after which a decline in the performance to around 85% correct classifications. This can be explained by the nature of Fatigue: when the Transition-to-Fatigue stage is reached the muscle is recruiting new MUAPs to cope with the load, therefore the classifier is classifying the Transition-to-Fatigue class with Non-fatigue. It can also be seen from the diagram that the classifier is stabilising at around 120 seconds to 85% correct classification without further significant decline or improvement. Moreover, although the classification performance declines, the automated system is still capable in performing exceptionally.

Table 3 shows the prediction of fatigue for the 5 subjects. The table shows the actual seconds until fatigue occurred and the predicted seconds until fatigue occurred. The error calculation is also shown in seconds and as percentage. The system was capable of predicting the onset of fatigue with an average error of 7.4 second over all subjects. It must be noted that the average duration of the actual trial is 174.20 seconds, from that we can represent the error as a percentage giving a 4.35% error. In realistic settings a 7.4 seconds or 4.35% error is acceptable when predicting the Fatigue onset.

## 7. Discussion

As the results show, this system has high classification accuracy. Indicating that the autonomous system is reliable when used by different subjects. As described in Section 3 this system has extracted the 1D spectro feature from the sEMG in real time, while previous studies using the same feature where conducted in an offline mode, for example [22], which used 10 subjects with similar experimental set-up. Each subject performed three trials, two trials where used for training the classifier and one for testing the classifier. That study showed the applicability of the 1-D spectro feature giving 85.72% correct classification on average of all subjects with a specificity of 98.4 % and sensitivity of 60.2% when classifying using LDA. The LDA has proven capable of successfully classifying the sEMG signals based on the training phase, and since the LDA is a method which is light on the resources, it was chosen as the most appropriate technique for this system. Table 4 shows a comparison in classification of the method used in this study (LDA) with others used in the literature. Nevertheless, in the future a more complex system could be developed from this prototype which may acquire a more sophisticated classification method although there is a possibility that it will take up too many resources and smooth running of the system will be effected. The labelling of the sEMG signal has been successfully used in other studies [21,22,34], meaning that this method is a promising way to ensure that the classification measures are correct.

The results demonstrate that the system is able to predict the time to fatigue, which means it will let the user know that fatigue will occur before the actual onset of fatigue. In this process, the Transition-to-Fatigue stage is very important. Albeit other researchers are using only two classes of fatigue, Non-Fatigue and Fatigue, previous research has shown that the Transition-to-Fatigue class can be identified by classification [21,32,33]. With this established finding, it is possible to predict the time to fatigue and not merely detect when the fatigue onset occurs. The system needs to be able to predict fatigue in order to achieve a proper autonomous system that will analyse the signals in real-time settings and will alert the user of possible fatigue before injury or unnecessary strain occurs. As this study shows, the system is capable of alerting the user in a simple way by using LED lights as indicators. In a future system, the indicators could be upgraded, e.g., the use of voice indicators.

## 8. Conclusion

In this study we have developed an autonomous system that is capable of predicting and detecting fatigue in real-time settings. With the use of the previously developed feature, 1D spectro, the sEMG signals were analysed and then successfully classified into fatigue classes using LDA, allowing the prediction of sEMG muscle fatigue. The mean classification accuracy tested on five individuals showed 90.37% with an error of 4.35% in predicting the time to when fatigue will onset. This paper has demonstrated the possibility to implement such a system for detecting/predicting localised muscle fatigue with exceptional accuracy bridging the clinical side with technical implementation.

## Figures and Tables

**Table 1.** Rule base for signal labelling.

Rules	IF Input 1 (Elbow Angle)	IF Input 2 (Angle Oscillation)	THEN Output
1	Non-Fatigue	Low	Non-Fatigue
2	Non-Fatigue	High	Transition-to-Fatigue
3	Transition-to-Fatigue	Low	Transition-to-Fatigue
4	Transition-to-Fatigue	High	Transition-to-Fatigue
5	Fatigue	Low	Fatigue
6	Fatigue	High	Fatigue

**Table 2.** Percent of Correct Classifications (Non-Fatigue and Transition-To-Fatigue).

Subject	Classification Accuracy
1	93.09
2	89.18
3	91.36
4	92.87
5	85.33
Mean	90.37
St.dev	3.22

**Table 3.** Time to Fatigue and its error calculation.

Subject	Actual seconds to Fatigue	Predicted seconds to Fatigue	Error in seconds	Error in % to Fatigue
1	185	189	4	2.16
2	153	144	9	5.88
3	192	186	6	3.13
4	179	188	9	5.03
5	162	171	9	5.56
Mean	174.20	175.60	7.40	4.35
St.dev	16.24	19.11	2.30	1.62

**Table 4.** Classification performance comparison between this study and other classification methods.

Classification Method	Accuracy on testing Set (in %)
LDA (This study)	90.3
Logistic Regression	84.8
Neural Network	80.4
Fuzzy K-Nearest Neighbours	82.6
OCAT Approach	89.1
